# 

**Published:** 1891-04

**Authors:** 


### CONTENTS FOR APRIL, 1891.

ORIGINAL COMMUNICATIONS

Muscular Atrophies: A Clinico-Pathological Study. By WILLIAM C. KRAUSS, M. D...... 513
A Second Note on Croupous Rhinitis. By FRANK HAMILTON POTTER, M. D.............. 525
Description of Photographic Plates of an Edematous Acardia........................ 527
The State Medical Examining and Licensing Board. By WILLIAM WARREN POTTER, M.D. 529

SOCIETY PROCEEDINGS.

New York Academy of Medicine—Section on Orthopedic Surgery........................ 532
Buffalo Pathological Society........................................................ 540

PERSONAL.

Dr. Edward N. Brush — Dr. Roswell Park................................................ 558

EDITORIAL.

Two Mischievous Bills........................................................ 559
The Medical Corps of the Empire State........................................ 561
An Amende Honorable.......................................................... 564
A Learned Judge.............................................................. 565
State Medical Library........................................................ 567
Rapid Transit Again.......................................................... 568
The Medical Department of the University of Buffalo.......................... 568
Street Cleaning in Large Cities............................................... 569

OBITUARY.

Dr. W. B. Sprague — Death of District Physician Vaughn........................ 570

BOOK REVIEWS.

A Text-Book of the Diseases of the Ear........................................ 571
A Compend of Equine Anatomy and Physiology.................................... 575
A Compend of Human Anatomy, Including the Anatomy of the Viscera.............. 576
The Essentials of Minor Surgery and Bandaging, with an Appendix on Venereal Diseases 576

